# Analysis of longitudinal coupling dynamic characteristics of deep sea mining vessel and stepped lifting pipe

**DOI:** 10.1038/s41598-021-98746-7

**Published:** 2021-09-28

**Authors:** QingHui Song, HaiYan Jiang, QingJun Song, LinJing Xiao, Yu Wang

**Affiliations:** 1 Department of Mechanical and Electronic Engineering, Shandong University of Science & Technology, Qingdao, 264005 China; 2 Tai-an School, Shandong University of Science & Technology, Tai-an, 271009 China

**Keywords:** Energy science and technology, Engineering

## Abstract

In deep-sea mining, the coupling dynamic response between the mining vessel and the lifting pipe is a significant problem, which directly affects the structural design of the lifting system and the safety of field operation. The characteristics of coupled motion model have not been fully considered in the existing research. Therefore, this paper uses time-domain coupled numerical model as the research object, considering ocean current, surface wave, pipe dynamics and vessel-pipe contact mechanics, to study the dynamic behavior of the lifting pipe and mining vessel during the process of deep-sea mining using AQWA and OrcaFlex softwares. The response amplitude operator (RAO) is used to compare the measured and simulations dynamic response of the mining vessel. There is a very good agreement in RAO between the experiments and simulations. The coupling simulation results show that the coupling effect has a significant effect on the time domain dynamic response of the lifting pipe, but has little effect on the average effective tension and longitudinal amplitude along the pipe length. The research results of this paper are of great significance to the safety design of deep-sea mining lifting system and the planning of deep-sea operation activities.

## Introduction

Deep sea contains a lot of resources, which can meet the needs of mankind for a long time in the future^1^. At present, the proven potential deep-sea mineral resources include polymetallic nodules, cobalt-rich crusts, and polymetallic sulfides, among which deep-sea manganese nodules and cobalt-rich iron-manganese crusts are abundant^2^. Marine solid mineral resources, such as the most common iron-manganese nodules, are usually found in the 4000–7000 m deep sea^3^. In order to extract these minerals from the deep sea bed, there are usually three methods, namely, seabed mechanical transportation, air lift system with ejector and hydraulic lift system with pump station^4^. Since 1970s, many research groups from the United States, Canada, China, France, Germany, Russia, Japan and India have studied the core technology of deep sea mining^5,6^. Among them, the pipeline transportation mining system is recognized as the most practical deep-sea mining system, and it is also the only deep-sea mining system that has been verified by deep-sea experiments^7^. The system typically consists of surface mining vessel, lifting pipe, lifting pump sets, buffer, lifting hose and subsea collector^6,8,9^. In this system, the lifting pipe must be equipped with pump sets for lifting the mineral resources to the vessel, and with a buffer to regulate the density of the ore-fluid mixture. On the other hand, the pump sets and the buffer also increase the stability of the lifting pipe^10^.

Due to the motion of the mining vessel, the lifting pipe is prone to longitudinal vibration, transverse vibration and torsional vibration^11^, and in this process, a large dynamic load is added to the lifting system^12^. At the same time, the vibration of the lifting pipe in turn affects the vessel motion. So, a complex coupling dynamic response system is formed between the pipe and the vessel. With the increase of the length of the pipeline, the nonlinear effect and damping effect of pipeline gradually increase, the top tension of pipeline gradually increases, and the coupling effect between mining vessel and pipeline becomes more obvious. For the vessel-pipe coupling system in the deep sea, Zan et al.^13^ proposed a coupled time-domain numerical model of pipelines and pipe-laying vessel motions. The model was solved by Newmark method and verified with OrcaFlex software. The results showed that there is a significant connection between the dynamic responses of pipelines and the pipe laying vessel motions. Wang et al.^14^ established the coupled dynamic model of deep-water flexible pipeline, carried out the hydrodynamic analysis of pipe laying vessel by using AQWA software, and completed the numerical simulation of flexible pipeline by using OrcaFlex software, and analyzed the factors affecting the dynamic characteristics of the pipeline. Dai and Liu^15^ combined the single body dynamic model of the mining vessel with the multi-body discrete element dynamic model of the lifting pipe, considered the heave motion and longitudinal and transverse towing motion caused by the sea wave, and established the overall dynamic model of the deep-sea mining system. Using multi-body dynamics simulation method and finite element method, Oh et al.^16^ designed a coupling device for a deep-seabed mining system, and analyzed the kinematic characteristics between the coupling device and the buffer system, flexible pipe and mining robot. Based on Morison’s equations, lumped mass method and three-dimensional potential flow theory, Sun et al.^17^ proposed a method of coupled vessel/riser/body system in deep sea mining combined with dynamic positioning (DP), and obtained the time domain simulation result of the vessel operated in two DP modes. Chung^18^ carried out offshore tests of deep-sea mining systems to measure the response of full-scale pipelines and the motion of mining vessel in the deep sea. The measurement results show that the axial stress may be one order of magnitude larger than the bending stress for a 5000 m long pipe, and the amplitude of the axial stress is coupled with the motion of the mining vessel. In order to improve the analysis efficiency, based on the subsystem synthesis method of deep-sea integrated mining system, Kim et al.^19^ established the motion equations of vessel-lifting pipe subsystem and the flexible pipe-mining robot subsystem respectively, and extended them to the comprehensive analysis of multiple mining robots.

The lifting pipe with a length of several kilometers is the weakest and most technically difficult part in the mining vessel-pipe coupling system. The safety and success of offshore mining mainly depends on the reliable design of the lifting pipe structure^4^. Therefore, the dynamic characteristics of the lifting pipe has always been a research hotspot. Erol^20^ established the dynamic model of the stepped lifting pipe, and studied the longitudinal vibration characteristics of the lifting system with and without dynamic vibration absorber (DVA) under the over damping and under damping modes respectively by using the method of separating variables. The exact analytical solutions of the free response and forced response of the lifting pipe under the heave motion of the mining vessel were obtained. Cheng et al.^21^ proposed a time-domain hybrid finite element–boundary element method to investigate nonlinear interaction between vessel waves and slender structures and obtained the dynamic characteristics of multi-component mooring lines and deep water steel catenary risers under wave action. Using the finite element and Wilson-θ direct integral methods, Liu and Xiao et al.^22,23^ simplified the mining pipeline as beam element, and established the physical model and mathematical model of the mining pipeline system. Based on the Lagrangian method and the lumped element method, Song et al.^24^ built a dynamic model of the rigid space stepped-pipe strings system and obtained the longitudinal vibration characteristic of the stepped lifting pipe with and without dynamic vibration absorbers.

As can be seen from the brief review of the most advanced research, the current research on deep-sea mining system mainly focuses on the modeling and simulation analysis of the whole deep-sea mining system cooperative operation, the dynamic characteristics analysis of the lifting pipe. However, there are few researches on the dynamic coupling model of the mining vessel and stepped lifting pipe as well as the effect of wave parameters on dynamic response of lifting pipe. Zhao et al.^25^ proposed that wave parameters have a significant effect on hydrodynamic response. Therefore, the main goal of this paper is to offer a qualitative evaluation of the influence of coupling effect on the dynamic characteristics of mining vessel and lifting pipe during deep-sea mining. Thus, a comprehensive vessel-pipe time-domain coupled model is established using the software OrcaFlex to simulate the dynamic responses of mining vessel and lifting pipe under different waves. Meanwhile, a 1:80 scale experimental model is used to carry out relevant tests in a wave pool to verify the coupling dynamic behavior. On the other hand, the influence of coupling force on dynamic characteristics of mining vessel and lifting pipe is sufficiently discussed using an illustrative example of a MAFUTA mining vessel.

### Description of testing conditions and model set-up

#### Description of testing conditions

The East Pacific Ocean seabed polymetallic nodules mining area is selected as the deep-sea mining operation area of this paper. The actual operation depth of deep sea mining is 5000–5300 m, the density of sea water is 1028 kg/m^3^. According to the measured data of sea state survey and referring to the Beaufort scale of winds^26^, the layout and recovery of the lifting pipe are considered as level 4 sea state, and the lifting operation is considered as level 6 sea state. The actual sea state parameters are shown in Table 1.

**Table 1:** Sea state parameters of deep sea mining.

Sea state level	Wind velocity (m/s)	Mean wave height (m)	Significant wave height (m)	Period (s)
4	8	2	2.5	8
6	16	4.9	6	10

The dynamic analysis of deep sea mining system involves current, waves, Reynolds number (Re), coupling effect and other factors. Among them, the current velocity changes obviously with the change of sea water depth. Using the computer fitting method^27^, the current velocity can be calculated as

1 $$ V_{c} = 0.1 + 1.6 \times \left( {\frac{5000 - z}{{5000}}} \right)^{12} $$

where *z* is the depth below ocean surface.

### Experimental model set-up

In the hydrodynamic model experiment of deep-sea mining vessel, the motion and force under the action of waves are mainly considered. Therefore, the Froude similarity rule of model and entity should be satisfied^28^, that is

2 $$ \frac{{V_{m} }}{{\sqrt {gL_{m} } }} = \frac{{V_{s} }}{{\sqrt {gL_{s} } }} $$

where,$V$ and $L$ are characteristic velocity and characteristic scale respectively, and subscripts *m* and *s* represent model and entity respectively.

At the same time, the motion and force of the mining vessel are periodic, and the period is *T*. So the Strouhal number of the model and the prototype should be equal^29^, and given by an equation of the form

3 $$ \frac{{V_{m} T_{m} }}{{L_{m} }} = \frac{{V_{s} T_{s} }}{{L_{s} }} $$

The coupling experiment of deep-sea mining vessel and lifting pipe was carried out at the National Offshore Technology Center (TianJin, China). The experimental pool is 130 m long, 18 m wide and 6 m deep and equipped with a moving platform and track, which can simulate various marine environments such as wind and waves. According to the size of experimental pool, the experimental model of deep-sea mining system is a 1:80-scale prototype. This paper takes the MAFUTA mining vessel as an example to analyze and determine the relevant parameters of the mining vessel model, as shown in Table 2.

**Table 2:** Main parameters of prototype and model of mining vessel.

Parameters	Length (m)	Moulded breadth (m)	Moulded depth (m)	Tonnage (ton)	Draft (m)	Moonpool diameter (m)
Prototype	169.5	25.7	12.1	10,905	4	8
Test model	2.12	0.321	0.151	0.0213	0.05	0.1

**Figure 1:**
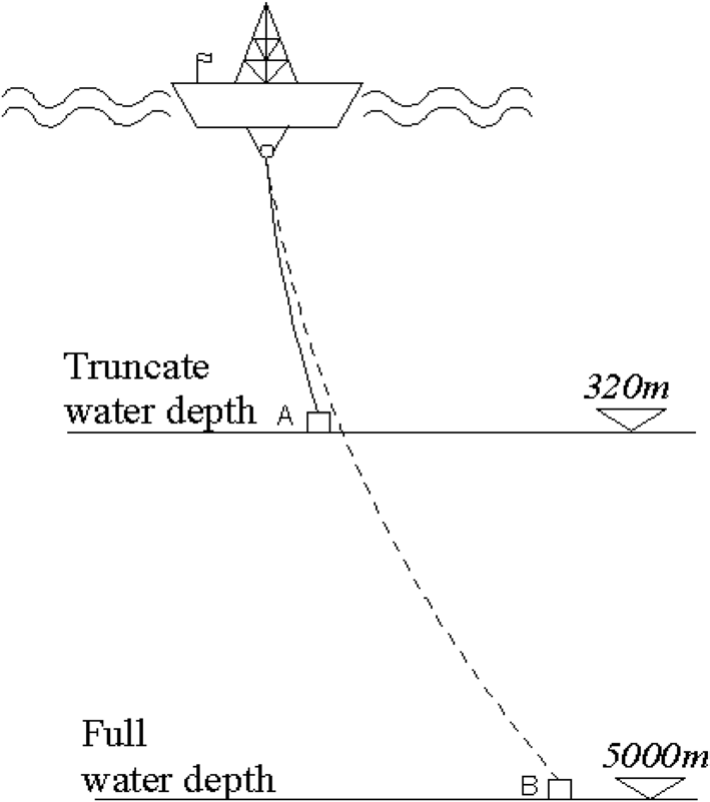
The truncated model of the testing system. Figure was created using AutoCAD 2020 software (URL: https://www.autodesk.com.cn/), and this image was drawn by the co-author QingHui Song.

The lifting pipe is a slender structure with engineering application depth of 5000 m and pipe diameter of about 224 mm. According to the common model scale, it is difficult to establish the model of lifting pipe. Therefore, the mixed model test technology based on the equivalent truncation design principle^30^ was used to build the lifting pipe model. The lifting pipe model is truncated according to the static similarity principle. In the truncation design, the truncation depth model and the full depth model should have the same layout and similar physical characteristics. Therefore, for a 5000 m mining lifting pipe, the corresponding truncated depth is 320 m, as shown in Fig. 1.

The lifting pipe consists of four parts, each of which has different physical characteristics. It is assumed that there is a buffer at the bottom of the lifting pipe with a concentrated mass of 30ton. Based on the similarity principle and truncation design criteria, the truncation model parameters and test model parameters of the lifting pipe are calculated as shown in Table 3.

**Table 3:** Parameters of model and entity of lifting pipe.

Parameters	Stepped section *i*	Length *L*_*i*_ (m)	Line mass *γ*_*i*_ (kg/m)	External diameter *D*_*i*_ (mm)	Cross-section area *S*_*i*_ (m^2^)	Elastic modulus *E*_*i*_ (Gpa)	Axial stiffness *E*_*i*_*S*_*i*_ (N)
Prototype	1	1000	331.95	254	0.0173	206.8	3.58 × 10^9^
	2	1000	227.98	240	0.0119	206.8	2.46 × 10^9^
	3	1500	171.21	232	0.0089	206.8	1.84 × 10^9^
	4	1500	129.9	226	0.0068	206.8	1.41 × 10^9^
Truncated model	/	320	3186.3	881.1	/	/	1.25 × 10^8^
Test model	/	4	0.5	11	/	/	244

**Figure 2:**
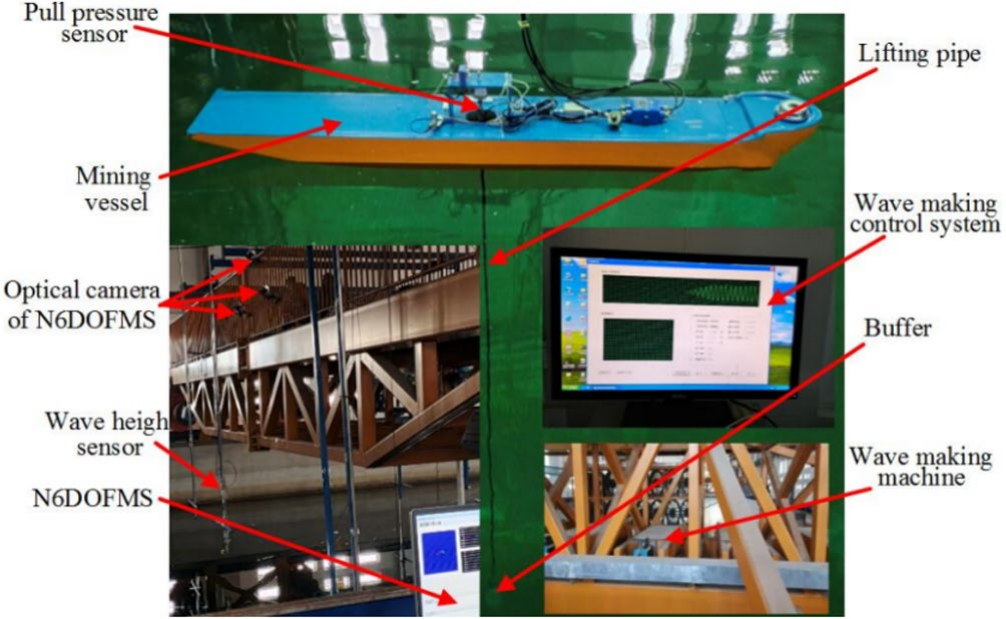
Coupling test model of deep sea mining vessel and lifting pipe (created by Microsoft Visio 2019 software, URL: https://www.microsoftstore.com.cn/visio/visio-standard-2019).

In the light of similarity criterion, the coupling test model of deep sea mining vessel and lifting pipe to be dealt with in the present study is shown in Fig. 2. A 0.05 kg steel ball is selected to replace the buffer of the lifting system in the experiment. Usually, the motion of the mining vessel in any space has 6 degrees of freedom (6-DOF), and the attitude information of the mining vessel is collected by three optical cameras, and the pull pressure sensor is installed at the junction of the mining vessel and the top of the lifting pipe to measure the coupling force, which is the irregular force generated at the vessel-pipe connection due to the coupling motion, that is, the tension between the mining vessel and the top of the lifting pipe^31^. Meanwhile, the information collection and analysis process was made by self-developed programs.

### Mechanical analysis of vessel-pipe coupled motion

In this paper, the vessel-pipe coupling system is studied numerically in time domain, and the motion equation of mining vessel is established by using the indirect time domain method from frequency domain to time domain, and the vibration equation of lifting pipe is established by using D'Alembert principle. Based on the analysis of mining vessel motion and the lifting pipe vibration, the elastic pipe vibration theory is used to calculate the vessel-pipe coupling force, and then the mathematical model of the vessel-pipe coupling motion is obtained.

### Equations of vessel-pipe coupled motion

In actual engineering, the coupling motion between the mining vessel and the lifting pipe is very complicated. At the coupling interface, the coupling effect between them can be regarded as the interaction between the vibration response of the lifting pipe and the vessel's motion response. Among them, the lifting pipe vibration is affected by inertial force, elastic restoring force and damping, and the mining vessel motion is affected by radiation force, static water restoring force, and wave force. In order to simplify the model, the following assumptions are proposed^27^:

1. The mining vessel is rigid body, the hull is stable and balanced in still water, only considering the heave and pitch motion of the vessel.
2. Waves acting on the vessel hull are deep-water small-amplitude waves, so the influence of shallow water waves and high-order nonlinear waves are ignored.
3. The stiffness of the hose connected at the bottom of the lifting pipe is very small, so its influence on the lifting pipe can be ignored, and the bottom of the lifting pipe is regarded as free end.
4. In the dynamic analysis, the bending and torsion deformation of the lifting pipe are ignored, and the lifting pipe is assumed to be a stepped-pipe string having four continuous parts with uniform and isotropic material.

**Figure 3:**
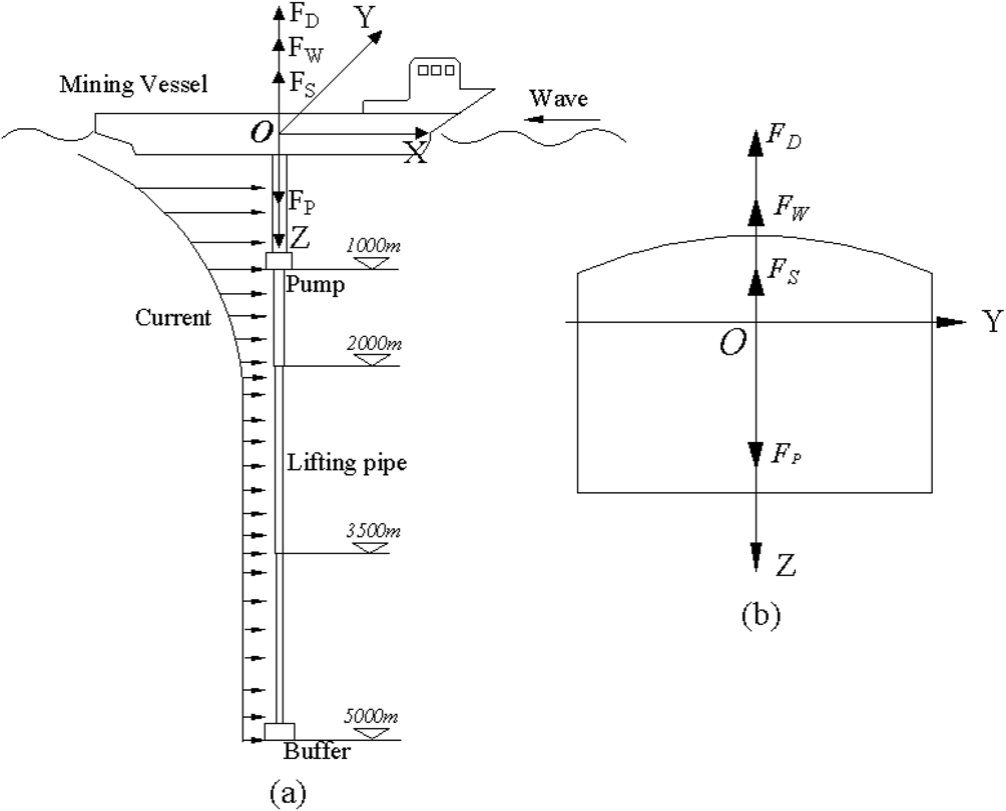
Mechanical model of vessel-pipe coupling system: (**a**) Model of mining system, (**b**) Section diagram of mining vessel (created by AutoCAD 2020 software,URL: https://www.autodesk.com.cn/).

Based on the above assumptions, the mechanical model of vessel-pipe coupling system is established, as shown in Fig. 3. $F_{S}$ is the restoring force in static water, $F_{W}$ is wave force, $F_{D}$ is radiation force, $F_{p}$ is the effective tension at the hinge position between the pipeline and the mining vessel, that is, the coupling force. The lifting pipe is vertical and a three-dimensional coordinate system *X*–*Y*–*Z* is introduced, where *X*–*Y* plane coincides with the still water surface, and *X*, *Y* and *Z* are the rotation axes of the vessel’s roll, pitch, and yaw, respectively.

In this paper, the potential flow theory is used to calculate the additional mass, damping and wave force of the mining vessel, and the frequency domain analysis of the coupled motion response between the vessel and the pipe has been made for the test model of the system when the vessel heaves and pitches. The frequency domain hydrodynamic parameters are transformed into the corresponding time domain hydrodynamic parameters by fast Fourier transform (FFT), and the dynamic characteristics of the vessel-pipe coupling motion are obtained. According to the principle of force balance, the dynamic equation of the system can be obtained as:

4 $$ M\ddot{X}(t) = F_{S} (t) + F_{W} (t) + F_{D} (t) + F_{P} (t) $$

where, *M* denotes the inertia matrix, *X*(*t*) represents the displacement vector, and* t* represents time.

### Motion of the mining vessel

Under the action of waves, the movement of mining vessels mainly includes surge, roll, sway, pitch, heave and yaw30, among them, vessel roll, swell and yaw can be eliminated by dynamic positioning. On the premise that roll can be completely compensated by heave compensation device, this paper studies the longitudinal coupling dynamic characteristics of deep sea mining vessel and lifting pipe.

Based on the potential flow theory, the section static water restoring force caused by the displacement change of mining vessel s given by

5 $$ F_{S} (t) = - CX(t) $$

where $C$ is the hydrostatic restoring matrix.

The wave force $F_{W} (t)$ of mining vessel can be calculated by convoluting the wave height time history function $\zeta (t)$ and impulse response function $g(\tau )$^32^, which gives

6 $$ F_{W} (t) = \int_{0}^{t} {g(\tau )} \zeta (t - \tau )d\tau $$

The impulse response function $g(\tau )$ can be obtained by the inverse Fourier transform of the first-order wave force transfer function $G(\omega )$ in the frequency domain, which represents the wave force generated by the simple harmonic wave of unit amplitude incident on the object. Then, the impulse response function $g(\tau )$ is

7 $$ g(\tau ) = \frac{1}{2\pi }\int_{ - \infty }^{\infty } {G(\omega )} e^{i\omega t} d\omega $$

The radiating force of the fluid is generated by the change of the surrounding fluid due to the motion of the vessel. In view of the linear wave approximation, the dynamic radiation of the ideal fluid in the time domain can be expressed as:

8 $$ F_{D} (t) = - A(\infty )\ddot{X}(t) - \int_{0}^{t} {K(t - \tau )} \dot{X}(\tau )d\tau $$

where,$A(\infty )$ represents the additional mass of the vessel at infinite frequency. The expression $\int_{0}^{t} {K(t - \tau )} \dot{X}(\tau )d\tau$ describes the fluid memory, which represents the memory effect of hydrodynamic reaction. The convolution term $K(t - \tau )$ is a matrix of retardation or memory functions containing the energy dissipation of the radiation waves generated by the vessel's motion.

According to Cummins pulse principle, the time delay function $K(t)$ in time domain can be obtained by transforming the time delay function in frequency domain into that in time domain through inverse Fourier transform:

9 $$ K(t) = \frac{2}{\pi }\int_{0}^{\infty } {B(\omega )} \cos \omega td\omega $$

where $B(\omega )$ represent the frequency dependent damping matrix.

After transforming the hydrodynamic coefficients of the mining vessel in frequency domain into the time domain, Eq. (4) takes the form

10 $$ \left( {M + A(\infty )} \right)\ddot{X}(t) + \int_{ - \infty }^{t} {\left[ {K(t - \tau )} \right]} \dot{X}(t)d\tau + CX(t) = F_{W} (t) $$

In numerical calculation, the term unrelated to the current velocity $\dot{X}(t)$ in the convolution part of the delay function is moved to the right end of the equation, and Eq. (10) is written in a more general form as follows:

11 $$ \tilde{A}\ddot{X}(t) + \tilde{B}\dot{X}(t)d\tau + \tilde{C}X(t) = \tilde{F}(t) $$

where,$\tilde{A}$ is the generalized mass matrix,$\tilde{B}$ is the damping matrix,$\tilde{C}$ is the hydrostatic restoring matrix,$\tilde{F}(t)$ is the sum of the memory effect part of the time delay function and other external forces.

### Motion of the lifting pipe

**Figure 4:**
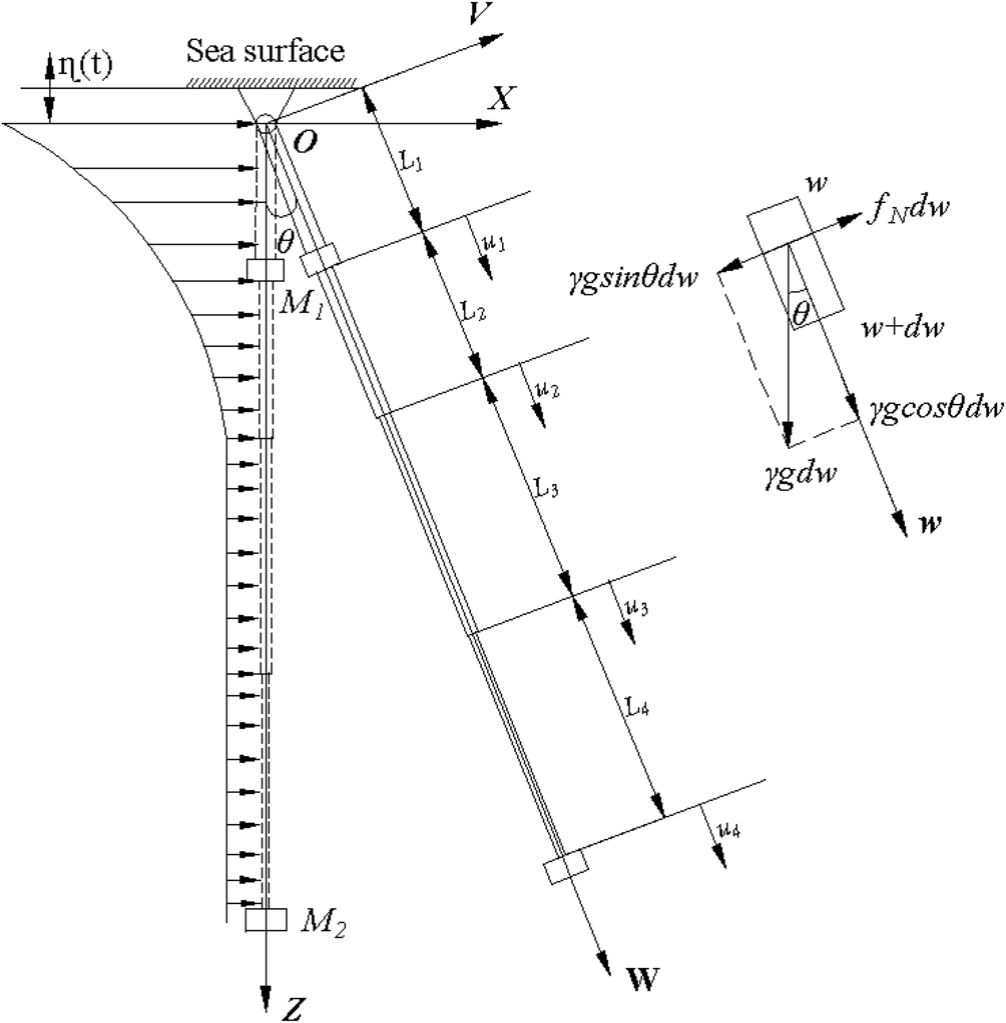
The mechanical model of the lifting pipe subsystem under the action of ocean current (created by AutoCAD 2020 software, URL: https://www.autodesk.com.cn/).

The stepped lifting pipe is composed of four-stage pipes, the top of which is hinged with the mining vessel and synchronized with the movement of the mining vessel, and the bottom of which is generally considered free, as shown in Fig. 4. The lifting pipe is equipped with a pump at L_1_ and a buffer at the end, as they are modeled by concentrated masses $M_{1}$ and $M_{2}$.Under the action of the ocean current, the lifter pipe will be laterally deviated by deflection angle $\theta$.

For small diameter and slender lifting pipe, the hydrodynamic force per unit length of the lifting pipe after offset can be calculated by Morrison equation^34,35^, we have that

12 $$ {\varvec{f}}_{{\varvec{N}}} = \frac{1}{2}C_{D} \rho D_{i} \left| {{\varvec{V}}_{{\varvec{N}}} } \right|{\varvec{V}}_{{\varvec{N}}} + \frac{1}{4}\pi C_{M} \rho D_{i}^{{\varvec{2}}} \dot{\user2{V}}_{{\varvec{N}}} $$

where,$C_{D}$ is the drag coefficient,$D_{i}$ is the outside diameter of the pipe,$\rho$ is the density of the sea water, $C_{M}$ is the inertial coefficient,${\varvec{V}}_{{\varvec{N}}}$ is the normal phase velocity component of ocean current velocity, ${\varvec{V}}_{{\varvec{N}}} = {\varvec{V}}_{C} \cos \theta$ and ${\varvec{V}}_{C}$ is the current velocity.

The displacement of any section *w* of the *i*-th pipe is a function of position and time, denoted as $u_{i} (w,t)$, and the equations of motion of the lifting pipe can be established as

13 $$ E_{i} S_{i} \frac{{\partial^{2} u_{i} }}{{\partial w^{2} }} - \gamma_{i} \frac{{\partial^{2} u_{i} }}{{\partial t^{2} }} - c_{i} \frac{{\partial u_{i} }}{\partial t} = 0 $$

where $c_{i}$ is external viscous damping coefficient.

The vibration differential Eq. (13) is solved by separating variables, and the solution of the vibration equation is assumed to be

14 $$ u_{i} (w,t) = \phi_{i} (w) \times e^{\lambda t} $$

where,$\phi_{i} (w)$ is the mode function of longitudinal vibration of pipeline,$e^{\lambda t}$ is the time function of pipeline motion,$\lambda$ is an eigenvalue of vibration differential equation,$\lambda = j\omega$,$j$ is an imaginary number,$\omega$ is the wave frequency.

Substituting (14) into Eq. (13) gives

15 $$ \left\{ {\begin{array}{*{20}c} {\frac{{d^{2} \phi_{i} (w)}}{{dw^{2} }} - \frac{{\gamma_{i} }}{{E_{i} S_{i} }}a_{i} \phi_{i} (w) = 0,} & {i = 1,2,3,4} \\ {\lambda^{2} + \frac{{c_{i} }}{{\gamma_{i} }}\lambda = a_{i} ,} & {i = 1,2,3,4} \\ \end{array} } \right. $$

where $a_{i}$ are complex constants to be determined.

Set $\upsilon_{{\text{i}}}^{2} { = }\frac{{\gamma_{i} }}{{E_{i} S_{i} }}a_{i}$, then the mode function of the stepped pipe can be expressed as

16 $$ \phi_{i} (w) = \overline{A}_{i} e^{{\upsilon_{i} w}} + \overline{B}_{i} e^{{ - \upsilon_{i} w}} \quad i = 1,2,3,4 $$

where $\overline{A}_{i}$ and $\overline{B}_{i}$ are complex constants that can be determined by the boundary conditions.

A harmonic motion with amplitude $\eta_{0}$ and frequency $\omega$ occurs in the mining vessel under waves, which is expressed as $\eta (t) = \eta_{0} e^{j\omega t}$, then the boundary and the continuity conditions of the lifting subsystem in the W–O-V coordinate system can be formulated as

17 $$ \left. {\begin{array}{*{20}c} {u_{1} (0,t){ = }\eta_{0} e^{j\omega t} \cos \theta } \\ {u_{i} (b_{i} ,t) = u_{i + 1} (b_{i} ,t)} \\ \end{array} } \right\} $$

where, *b*_*i*_ are introduced variables,$b_{i} = \sum\nolimits_{j = 1}^{i} {L_{j} } \quad (i = 1,2,3,4)$.

According to the condition of force continuity, the axial force of the second stage lifting pipe is equal to the sum of the first stage’s axial force and the pump set’s inertia force. At the junction of the third pipe and the fourth pipe, the axial force of the two pipes is equal, and the sum of the axial force and inertia force at the end of the fourth pipe is zero. This means that

18 $$ \left. {\begin{array}{*{20}c} {\left. {E_{1} S_{1} \frac{{\partial u_{1} (x,t)}}{\partial x}} \right|_{{x = b_{1} }} + \left. {M_{1} \frac{{\partial^{2} u_{1} (x,t)}}{{\partial t^{2} }}} \right|_{{x = b_{1} }} = \left. {E_{2} S_{2} \frac{{\partial u_{2} (x,t)}}{\partial x}} \right|_{{x = b_{1} }} } \\ {\left. {E_{i} S_{i} \frac{{\partial u_{i} (x,t)}}{\partial x}} \right|_{{x = b_{i} }} = \left. {E_{i + 1} S_{i + 1} \frac{{\partial u_{i + 1} (x,t)}}{\partial x}} \right|_{{x = b_{i} }} ,i = 2,3} \\ {\left. {E_{4} S_{4} \frac{{\partial u_{4} (x,t)}}{\partial x}} \right|_{{x = b_{4} }} + \left. {M_{2} \frac{{\partial^{2} u_{4} (x,t)}}{{\partial t^{2} }}} \right|_{{x = b_{4} }} = 0} \\ \end{array} } \right\} $$

Substituting the modal function Eq. (16) and the substitution of the expression $\lambda = j\Omega$ into the boundary conditions Eq. (17) and continuity conditions Eq. (18), the following equation can be obtained

19 $$ {\mathbf{G}} \times [\overline{{A_{1} }} ,\overline{{B_{1} }} ,\overline{{A_{2} }} ,\overline{{B_{2} }} ,\overline{{A_{3} }} ,\overline{{B_{3} }} \overline{{A_{4} }} ,\overline{{B_{4} }} ]^{T} = [\eta_{0} \cos \theta ,0,0,0,0,0,0,0]^{T} $$

where

$$ {\mathbf{G}} = \left[ {\begin{array}{*{20}l} 1 \hfill & 1 \hfill & 0 \hfill & 0 \hfill & 0 \hfill & 0 \hfill & 0 \hfill & 0 \hfill \\ {e^{{\upsilon_{1} b_{1} }} } \hfill & {e^{{ - \upsilon_{1} b_{1} }} } \hfill & { - e^{{\upsilon_{2} b_{1} }} } \hfill & { - e^{{ - \upsilon_{2} b_{1} }} } \hfill & 0 \hfill & 0 \hfill & 0 \hfill & 0 \hfill \\ {\alpha_{1} e^{{\upsilon_{1} b_{1} }} } \hfill & {\beta_{1} e^{{ - \upsilon_{1} b_{1} }} } \hfill & { - \kappa_{2} e^{{\upsilon_{2} b_{1} }} } \hfill & {\kappa_{2} e^{{ - \upsilon_{2} b_{1} }} } \hfill & 0 \hfill & 0 \hfill & 0 \hfill & 0 \hfill \\ 0 \hfill & 0 \hfill & {e^{{\upsilon_{2} b_{2} }} } \hfill & {e^{{ - \upsilon_{2} b_{2} }} } \hfill & { - e^{{\upsilon_{3} b_{2} }} } \hfill & { - e^{{ - \upsilon_{3} b_{2} }} } \hfill & 0 \hfill & 0 \hfill \\ 0 \hfill & 0 \hfill & {\kappa_{2} e^{{\upsilon_{2} b_{2} }} } \hfill & { - \kappa_{2} e^{{ - \upsilon_{2} b_{2} }} } \hfill & { - \kappa_{3} e^{{\upsilon_{3} b_{2} }} } \hfill & {\kappa_{3} e^{{ - \upsilon_{3} b_{2} }} } \hfill & 0 \hfill & 0 \hfill \\ 0 \hfill & 0 \hfill & 0 \hfill & 0 \hfill & {e^{{\upsilon_{3} b_{3} }} } \hfill & {e^{{ - \upsilon_{3} b_{3} }} } \hfill & { - e^{{\upsilon_{4} b_{3} }} } \hfill & { - e^{{ - \upsilon_{4} b_{3} }} } \hfill \\ 0 \hfill & 0 \hfill & 0 \hfill & 0 \hfill & {\kappa_{3} e^{{\upsilon_{3} b_{3} }} } \hfill & { - \kappa_{3} e^{{ - \upsilon_{3} b_{3} }} } \hfill & { - \kappa_{4} e^{{\upsilon_{4} b_{3} }} } \hfill & {\kappa_{4} e^{{ - \upsilon_{4} b_{3} }} } \hfill \\ 0 \hfill & 0 \hfill & 0 \hfill & 0 \hfill & 0 \hfill & 0 \hfill & {\alpha_{4} e^{{\upsilon_{4} b_{4} }} } \hfill & {\beta_{4} e^{{ - \upsilon_{4} b_{4} }} } \hfill \\ \end{array} } \right] $$

Note that the coefficients $\alpha_{i}$,$\beta_{i}$ and $\kappa_{i}$ are the expressions of *M*_*i*_, *E*_*i*_, *S*_*i*_,$\upsilon_{i}$ and $\lambda$, as follows

20 $$ \left. \begin{gathered} \alpha_{1} = M_{1} \lambda^{2} + E_{1} S_{1} \upsilon_{1} \hfill \\ \alpha_{4} = M_{2} \lambda^{2} + E_{4} S_{4} \upsilon_{4} \hfill \\ \beta_{1} = M_{1} \lambda^{2} - E_{1} S_{1} \upsilon_{1} \hfill \\ \beta_{4} = M_{2} \lambda^{2} - E_{4} S_{4} \upsilon_{4} \hfill \\ \kappa_{i} = E_{i} S_{i} \upsilon_{i} \quad i = 2,3,4 \hfill \\ \end{gathered} \right\} $$

In the environment of Matlab R2020, the program (code) is written to model the longitudinal vibration of the pipe of deep-sea mining system, and the complex constants $\overline{A}_{i}$ and $\overline{B}_{i}$ are obtained. Then the forced longitudinal vibration displacement of the lifting pipe can be obtained.

### Coupling force

The total axial load of the pipe is composed of the gravity load caused by its own weight and the dynamic load caused by the vibration of the mining vessel. The axial dynamic load of any section *w* can be obtained by the following equation:

21 $$ F_{di} (w,t) = E_{i} S_{i} \frac{{\partial u_{i} (w,t)}}{\partial w} $$

Then we can get the dynamic coupling load between the mining vessel and the pipeline as follow

22 $$ F_{d1} (0,t) = E_{1} S_{1} \left. {\frac{{\partial u_{1} (w,t)}}{\partial w}} \right|_{w = 0} = E_{1} S_{1} q(t)\left( {\overline{A}_{1} \upsilon_{1} - \overline{B}_{1} \upsilon_{1} } \right) $$

Therefore, the coupling force $F_{p}$ between the pipe and the mining vessel considering gravity is as follows

23 $$ F_{p} (t) = \, E_{1} S_{1} q(t)\left( {\overline{A}_{1} \upsilon_{1} - \overline{B}_{1} \upsilon_{1} } \right) + \left[ {M_{1} + M_{2} + \gamma_{1} L_{1} + \gamma_{2} L_{2} + \gamma_{3} L_{3} + \gamma_{4} L_{4} } \right]g\cos \theta $$

It can be seen from the coupling force Eq. (23) that the coupling force is determined by the structure of the lifting pipe, the damping coefficient, and the motion response of the mining vessel.

**Figure 5:**
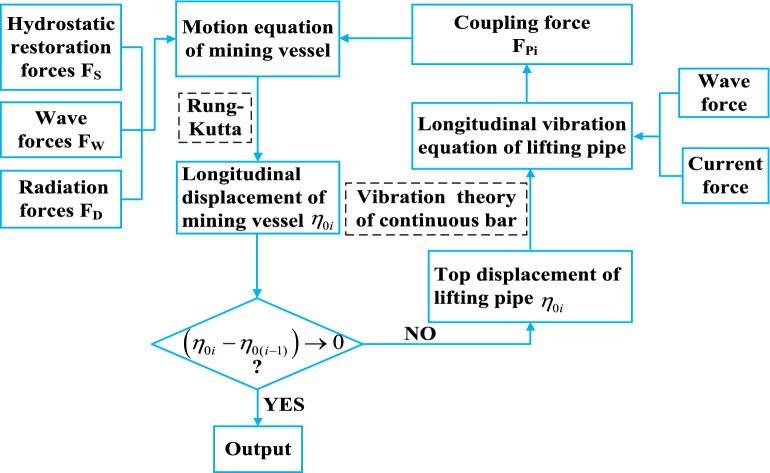
Flow chart of numerical calculation of coupled model (created by Microsoft Visio 2019 software, URL: https://www.microsoftstore.com.cn/visio/visio-standard-2019).

The coupling between the mining vessel and the lifting pipe system is realized by setting boundary conditions at their connecting positions. Because the vessel and the pipe are hinged, there is the same displacement and force in their connection position, and no bending moment occurs. The calculation process of coupling model is shown in Fig. 5.

### Analysis and verification

#### RAO prediction of the mining vessel

**Figure 6:**
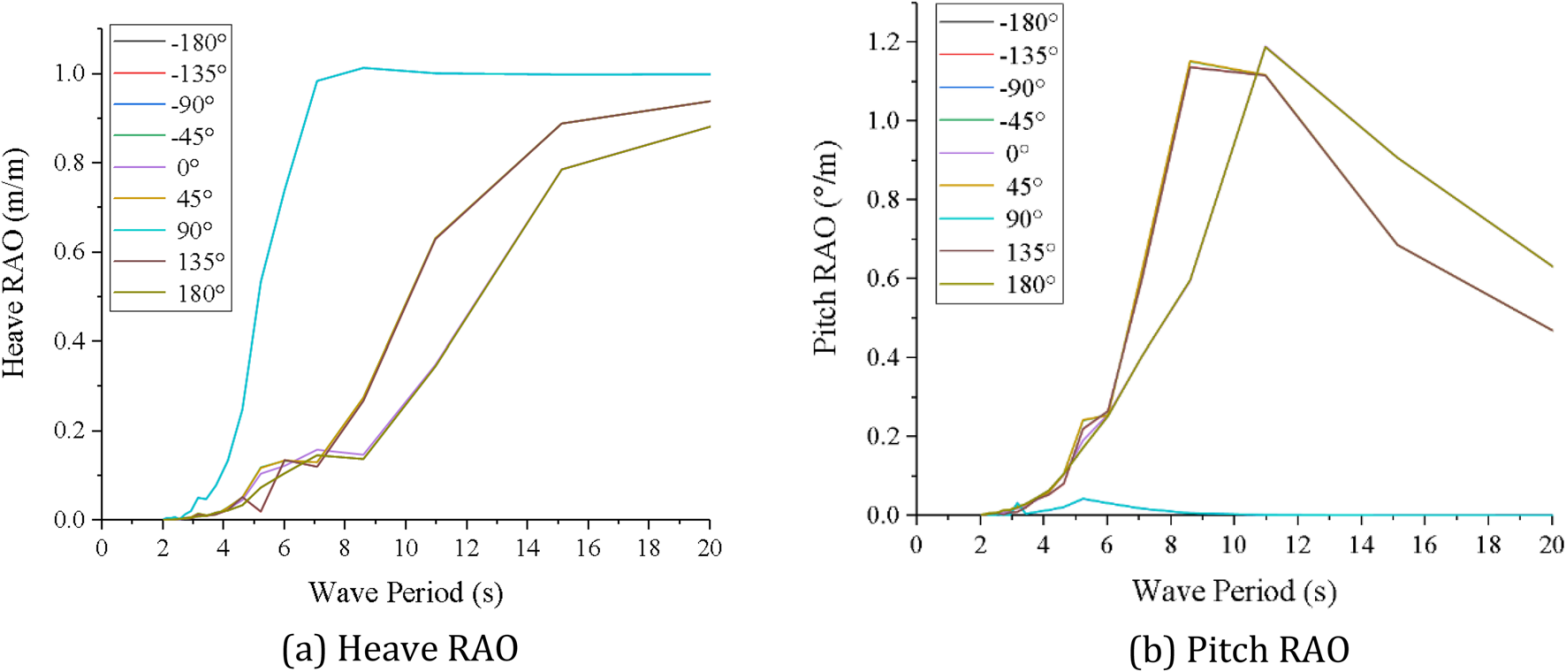
RAO curves of mining vessel at different wave directions. (**a**) Heave RAO, (**b**) Pitch RAO (created by Origin 2017 software, URL: https://www.originlab.com/).

In this paper, the MAFUTA mining vessel is taken as an example, and the boundary element method AQWA is used to analyze the motion of mining vessel, and to predict the response of the mining vessel motion under waves. The software covers the full range of fluid analysis, including transition analysis and response amplitude operators (RAOs) for the calculation of coupling dynamics. Each displacement RAO consists of a pair of numbers that define the vessel response for one particular wave direction and period^14^. The two numbers refer to the amplitude which relates the amplitude of vessel motion to the amplitude of wave, and the phase which defines the timing of vessel motion relative to the wave, respectively^36^. During the simulation analysis of mining vessel motion, the wave frequency range is 0.05 Hz ~ 0.5 Hz, and the wave direction range is − 180° ~ 180°. RAO is calculated once every 45° in the wave direction range. The simulation results are shown in Fig. 6.

As can be seen from Fig. 6, when the wave direction is 90° and the wave period is 8.68 s, the heave RAO reaches the maximum value of 1.01 m/m; when the wave direction is 180° and the wave period is 11* s*, the pitch RAO reaches the maximum of 1.19°/m. For heave and pitch motions, the RAO curves of the mining vessel under the action of the symmetrical wave directions are basically the same, indicating that symmetric mining vessel does not significantly affect motion in horizontal directions. Therefore, the RAO curves of 90°, 135° and 180° wave directions can be used to describe the heave and pitch motions of the mining vessel at the above nine different directions. Meanwhile, the RAO curves at 135° and 180° wave directions are similar, so we choose the wave directions at 90° and 180° as the representative for the dynamic analysis of deep sea mining system.

Several frequency values are selected near the resonance frequencies of heave and pitch motions of mining vessels, and the RAO regular wave experiments are carried out at 90° and 180° wave directions. The motion parameters of the vessel model are measured in real time by self-developed programs, and the RAO value under the experimental conditions can be calculated by the following formula

24 $$ RAO = \frac{{\eta_{0i} }}{{\zeta_{i} }} $$

where, $\eta_{0i}$ refers to the amplitude of response for the *i*-th degree of freedom (i.e., heave, pitch) and $\zeta_{i}$ refers to the incident regular wave amplitude.

**Figure 7:**
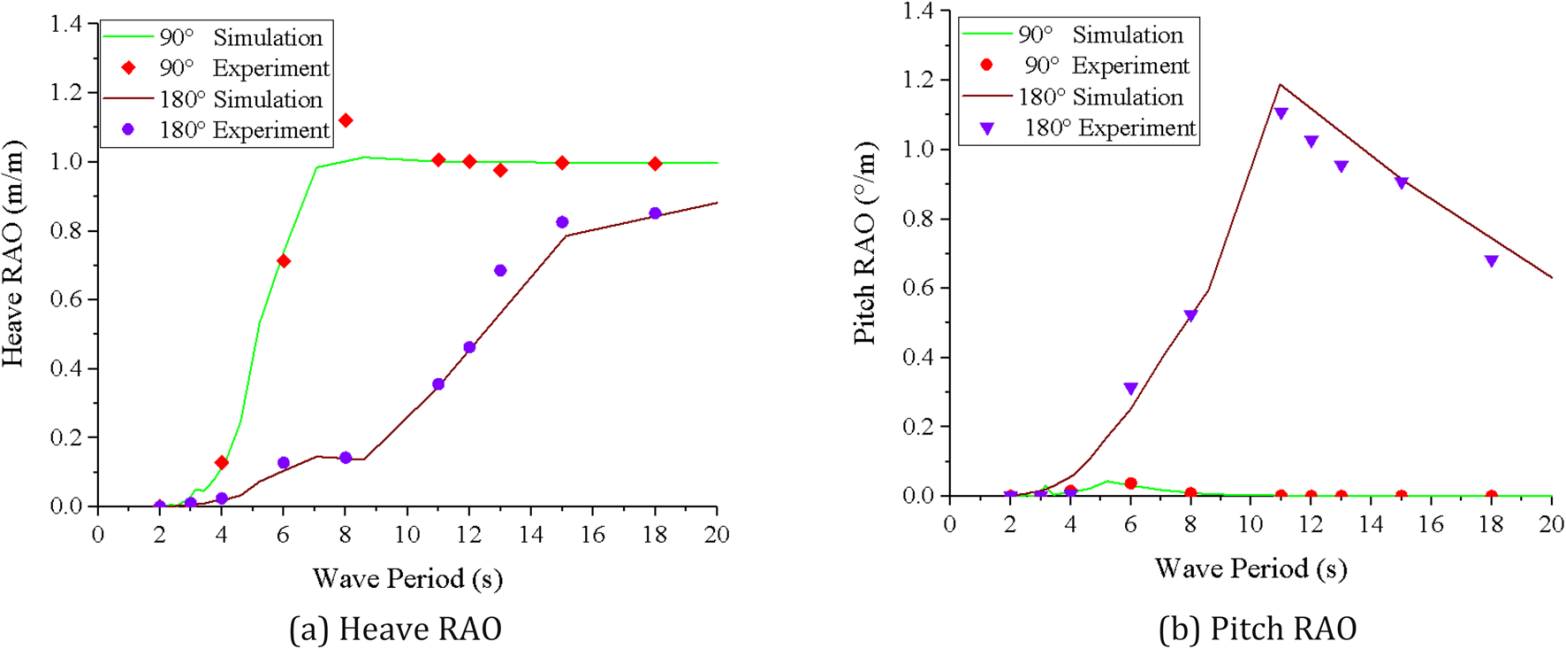
Simulation and experimental results of RAO. (**a**) Heave RAO, (**b**) Pitch RAO (created by Origin 2017 software, URL: https://www.originlab.com/).

It is assumed that the mining vessel is considered to be dynamically stable under the action of waves, the comparison between the experimental RAO and the simulated RAO is conducted on aspects of regular waves. As shown in Fig. 7, the trends of RAOs obtained from the experiment were found to agree with that obtained from the FAST coupled simulations using AQWA. However, there are some differences between them in the high frequency band, which is mainly due to the small excitation energy of the high frequency wave, resulting in the small motion response of the mining vessel. In addition, in each experiment, the wave surface is not completely stable, so it is difficult to obtain accurate measurements.

### Analysis of the dynamic characteristics

**Figure 8:**
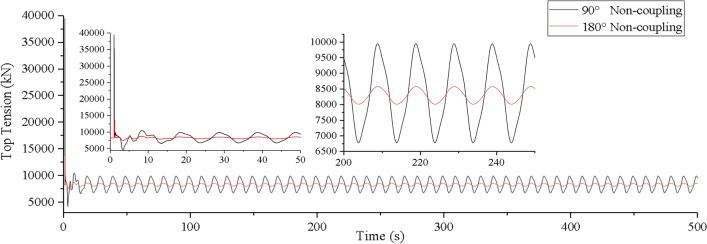
Time history curve of top tension of lifting pipe (created by Origin 2017 software, URL: https://www.originlab.com/).

**Figure 9:**
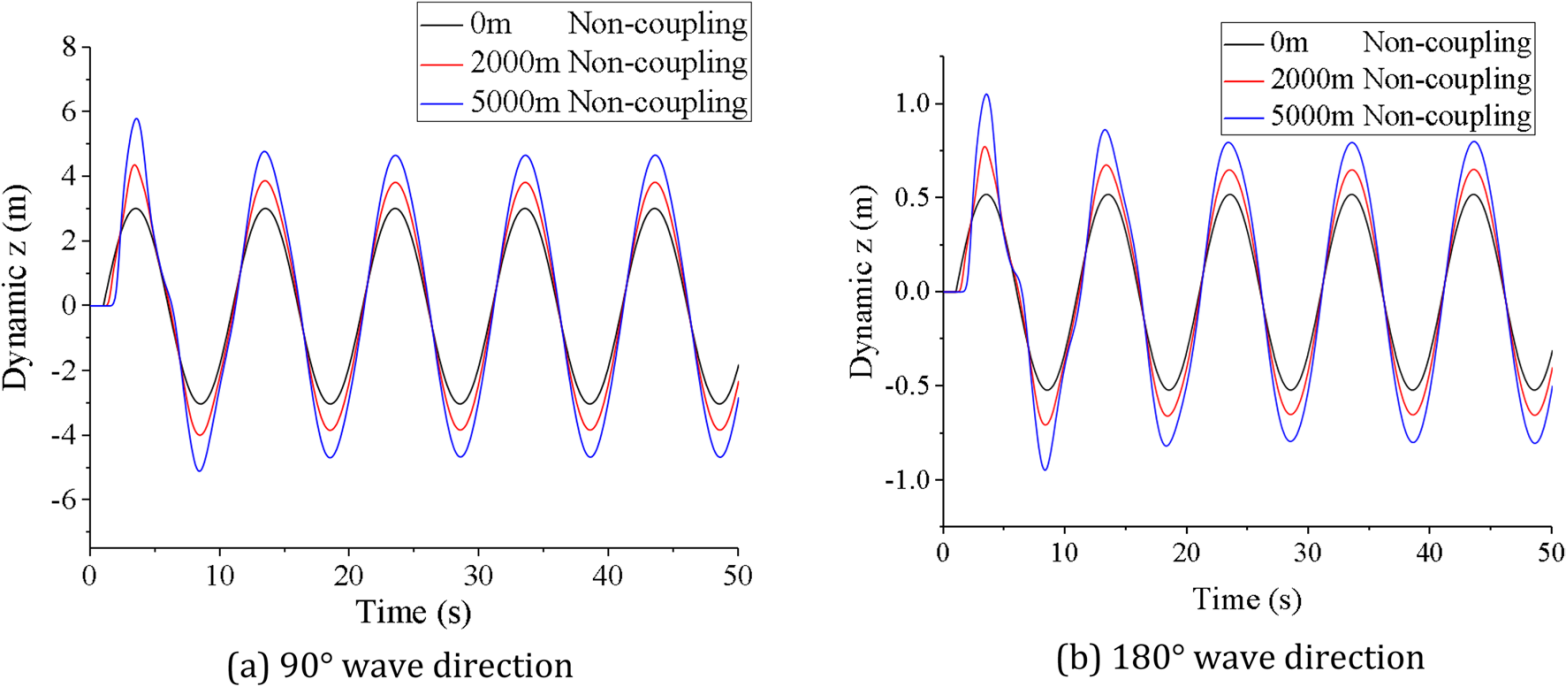
Longitudinal vibration displacements of lifting pipe (created by Origin 2017 software, URL: https://www.originlab.com/).

Assuming that the lifting system is operating under the waves, the motion of the mining vessel affects the vibration displacement and dynamic load of the lifting pipe. During lifting operation, the sea state generally does not exceed level 6. The longitudinal motion of the mining vessel under waves is simulated by applying sinusoidal excitation on the upper end of the lifting pipe model. Through the simulation analysis, the vibration displacement and top tension of lifting pipe under level 6 sea state are obtained without considering the vessel-pipe coupling factor, as shown in Figs. 8 and 9.

It can be seen from Figs. 8 and 9, when the coupling factor between vessel and pipe is not considered, the top tension of the lifting pipe has a transient step response at the initial vibration, and it enters into a steady-state constant amplitude oscillation process after about 5 s. At this time, the oscillation frequency of the top tension is the same as the frequency of the mining vessel motion. The irregular oscillation time of the top tension is basically the same along the action of two different wave directions. After reaching the steady state, the equilibrium position of the top is the initial position of the top of the pipe without external harmonic excitation. Compared with 180° wave direction, the longitudinal vibration response of the lifting pipe is more significant at 90° wave direction. Under the two wave directions, the maximum steady-state longitudinal vibration amplitude of the lifting pipe is 4.88* m* and 0.81* m* respectively, with a difference of 83.4%. The maximum steady-state top tension value is 9947.26kN and 8579.77kN respectively, with a difference of 13.75%.

### Analysis of coupling dynamic characteristics

**Figure 10:**
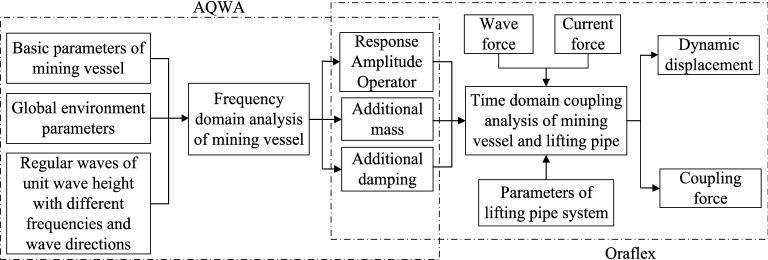
Time domain coupling simulation process of deep sea mining system (created by Microsoft Visio 2019 software, URL: https://www.microsoftstore.com.cn/visio/visio-standard-2019).

The time domain simulation of mining vessel-lifting pipe coupling motion was carried out by using the AQWA software and OrcaFlex software. In Fig. 10, the flowchart of this algorithm is presented. Firstly, the mining vessel motions under different regular waves are simulated by using AQWA software, and obtain the data of 6DOF RAO, additional mass and damping. Then, the data is imported into OrcaFlex software, and the model of lifting system is established in OrcaFlex. Finally, the time-domain coupling analysis of mining vessel and lifting pipe is carried out by OrcaFlex under different environmental parameters such as wave, ocean current, etc., and obtain the coupling dynamic responses of the system.

**Figure 11:**
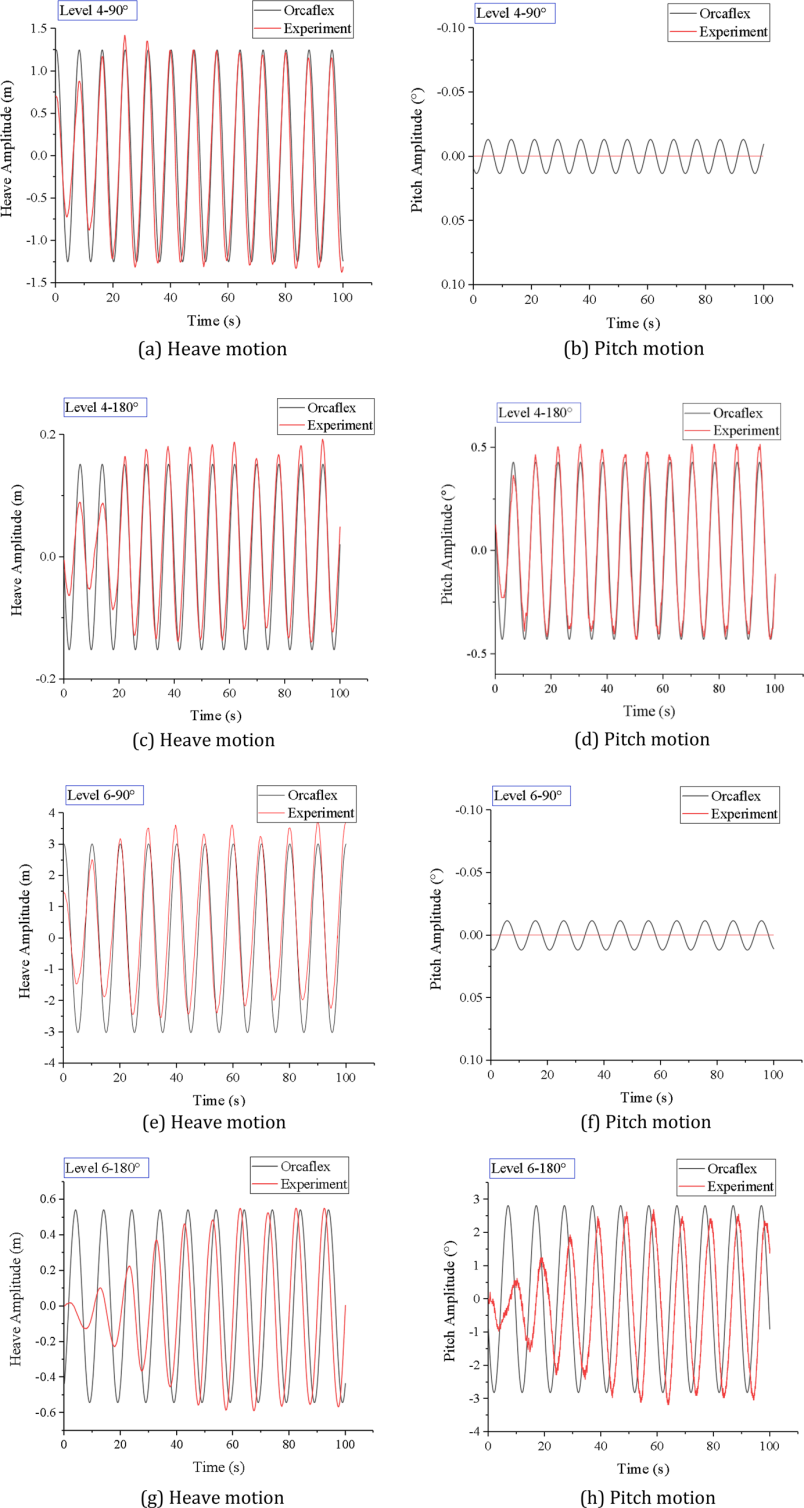
The mining vessel motion at experiment and simulation conditions (created by Origin 2017 software, URL: https://www.originlab.com/).

We first time make experiment on the coupling action of the lifting pipe on the vessel motion. When the mining vessel is equipped with a lifting pipe, the current force, pipeline restoring force, damping force and inertia force on the lifting pipe will affect the motion of the mining vessel. By using OrcaFlex software, the heave and pitch motion of the mining vessel with lifting pipe under specific sea states and wave directions are simulated and analyzed. Meanwhile, the 6-DOF motion experiments of the mining vessel model with lifting pipe are carried out. The results of experiment and simulation are shown in Fig. 11.

Figure 11 shows that the experimental results of heave/pitch motion of the mining vessel with lifting pipe are basically consistent with the simulation results, and the amplitudes of the mining vessel motion under different sea states are shown in Table 4. It can be found that the wave direction and sea state level have a significant action on the amplitude of the mining vessel motion. The heave amplitude at 90° wave direction is greater than that at 180° wave direction, and the difference is 82.78% when the sea state is level 6. The pitch amplitude at 180° wave direction is greater than that at 90° wave direction, and the difference is 99.64% when the sea state is level 6. The amplitude of the mining vessel motion with level 6 sea state is greater than that with level 4 sea state, the difference of heave amplitude is 76.15% at 180° wave direction. It is not difficult to find that the heave motion of the mining vessel is significant when the wave direction is 90°, while the pitch motion of the mining vessel is significant when the wave direction is 180°.

**Table 4:** The amplitude of mining vessel motion under different conditions.

Wave Direction	Sea state level	Heave Amplitude (m)			Pitch Amplitude (°)		
		Vessel	Vessel + pipe	Decrement	Vessel	Vessel + pipe	Decrement
90°	4	1.25	1.25	0	0.01	0.01	0
	6	3.02	3.02	0	0.01	0.01	0
180°	4	0.18	0.124	31.11%	0.65	0.43	33.85%
	6	0.78	0.52	33.33%	2.85	2.81	1.40%

**Figure 12:**
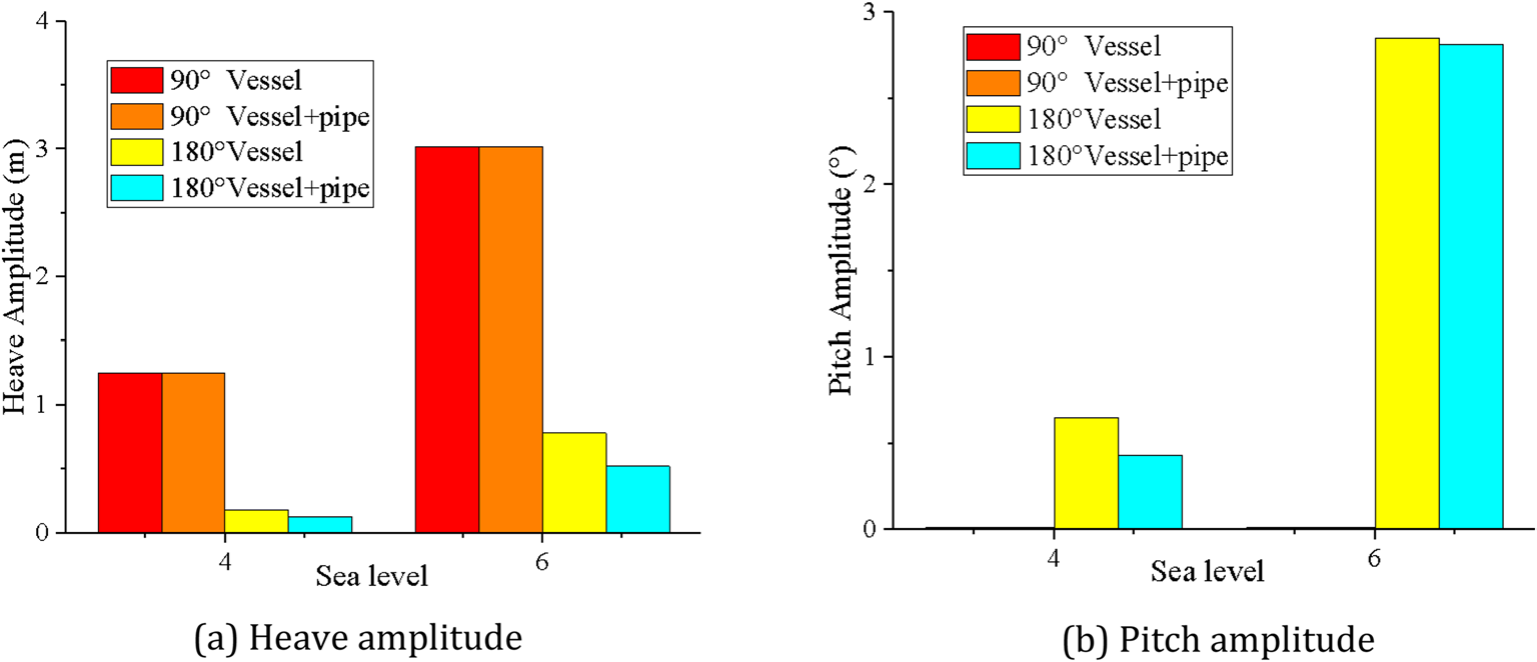
Heave amplitude and pitch amplitude of mining vessel with or without lifting pipe under different sea states (created by Origin 2017 software, URL: https://www.originlab.com/).

By comparing the motion amplitude of the mining vessel before and after the additional lifting pipe in Table 4, it can be found that the amplitude decreases at 180° wave direction, and basically remains unchanged at 90° wave direction, which indicates that the influence of the lifting pipe on the amplitude of the mining vessel motion is closely related to the wave direction. When the wave direction is 180° and the lifting pipe is attached, the heave amplitude and pitch amplitude of the mining vessel under level 4 and level 6 sea states will decrease by 31.11%, 33.33%, 33.85% and 1.4%, respectively, as shown in Fig. 12. The wave period is similar to the natural period of the pitching of the mining vessel in the fourth level sea state, resulting in the increase of the pitching amplitude of the mining vessel under level 4 sea state. After the lifting pipe is added to the mining vessel, the natural frequency of the system changes, and the pitching amplitude of the mining vessel decreases greatly, close to 33.85%. Under level 6 sea state, the wave period is far away from the natural period of pitching of the mining vessel. At the same time, because the lifting pipe is installed at the center of gravity of the mining vessel, the lifting pipe has little impact on the pitching amplitude of the mining vessel, which is only 1.40%.

**Figure 13:**
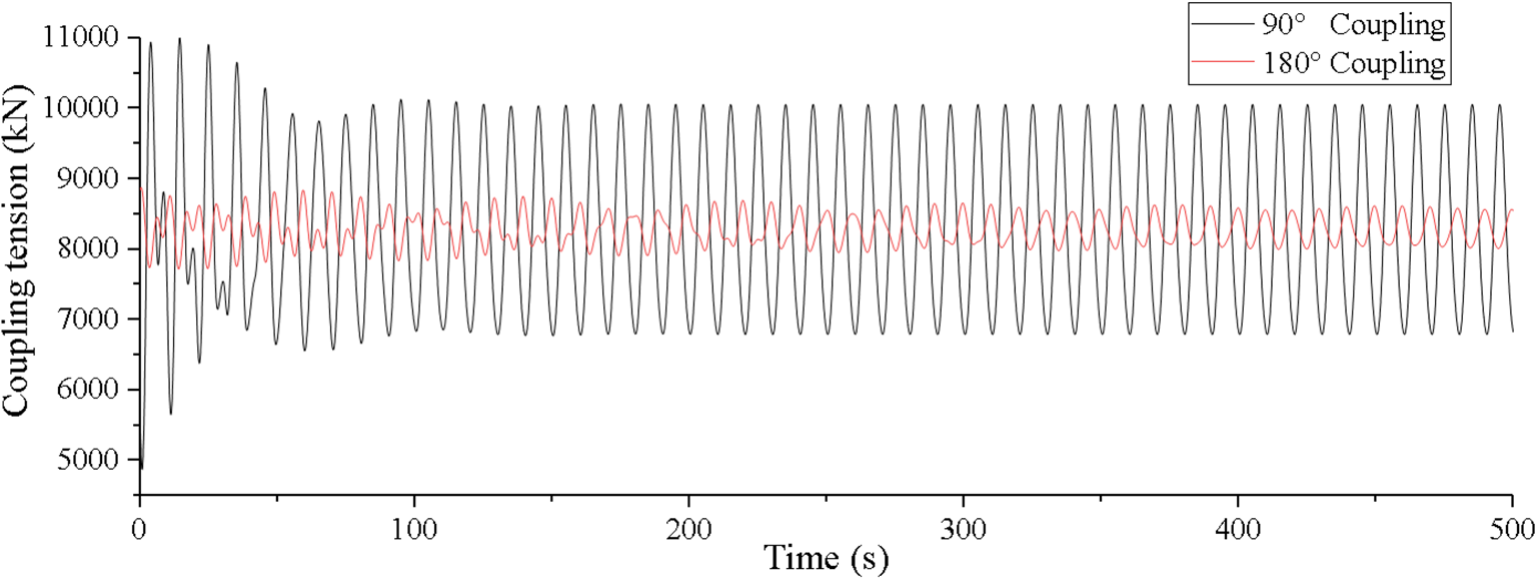
Time history of coupling tension under level 6 sea state on OrcaFlex (created by Origin 2017 software, URL: https://www.originlab.com/).

**Figure 14:**
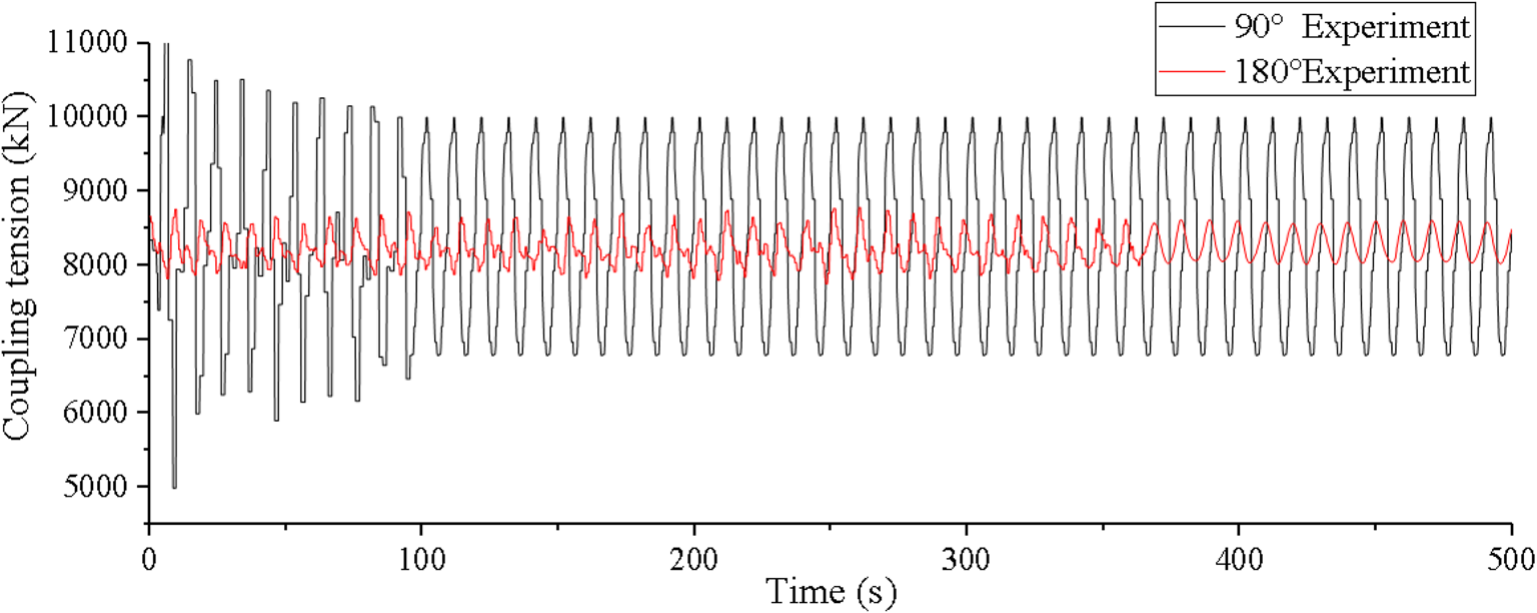
Time history of coupling tension under level 6 sea state in experiment (created by Origin 2017 software, URL: https://www.originlab.com/).

**Figure 15:**
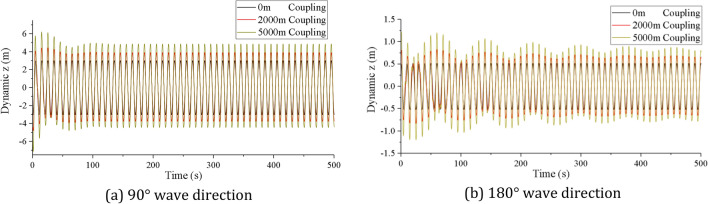
Time history of longitudinal vibration displacements of lifting pipe (created by Origin 2017 software, URL: https://www.originlab.com/).

Secondly, we make experiment on the coupling action of the vessel motion on the dynamic response of the lifting pipe. The coupling action of mining vessel motion on the lifting pipe is mainly reflected in the top tension and vibration displacement of the lifting pipe. Orcaflex software is used to simulate the coupling tension and vibration displacement under the condition of level 6 sea state with 90°and 180° wave directions, and the coupling tension is verified by experiments, as shown in Figs. 13, 14, and 15.

It can be seen from Fig. 13 that if coupling action is taken into account, the lifting pipe top tension has two states: coupling oscillation and steady oscillation. In the coupling oscillation stage, the wave direction has a greater influence on the coupling tension value and the coupling action time. The coupling action time is shorter and the coupling tension value is greater at 90° wave direction. When the wave directions are 180° and 90°, the coupling action time is 300 s and 100 s respectively, the difference is 66.67%, the maximum coupling tension value is 8880.6kN and 11,005.6kN, the difference is 19.31%. In the steady oscillation stage, the amplitude, period and equilibrium position of the tension oscillation are basically consistent with the top tension of the lifting pipe without considering the coupling factor (as shown in Fig. 8). The maximum steady tension values under different wave directions are 8585.2kN and 10,058.8kN respectively, which are reduced by 3.33% and 8.6% compared with the maximum coupling tension values.

Considering Figs. 8, 13, and 14, it can be found that the test value is closer to the simulation value of the top tension considering the coupling action. However, in the stage of coupling, the irregular oscillation trend of the experimental value and the simulated value is not exactly the same, which is mainly caused by the equivalent truncation design of the experimental model of the lifting pipe. This result has little effect on the coupling characteristic analysis of the system and the safety design of the pipeline, and its influence can be ignored.

As shown in Fig. 15, the longitudinal vibration displacement of the lifting pipe also has two stages of coupling oscillation and steady-state oscillation after considering the coupling action. In the coupling oscillation stage, the wave direction has a great influence on the coupling vibration amplitude and coupling action time. The coupling vibration amplitude is larger at 90° wave direction, and the coupling action time is longer at 180° wave direction. The difference between the maximum coupling vibration displacement and the maximum steady vibration displacement under different wave directions are 27.4% and 31.3% respectively. At the same wave direction, the coupling action time of vibration is not the same along the pipe length, showing a trend that the upper end is smaller and the lower end is larger. Similarly, in the stead oscillation phase, the amplitude, vibration period and equilibrium position are basically the same as those in Fig. 9. In addition, combining Figs. 13 and 15, it can be seen that the coupling vibration law of the bottom displacement of the lifting pipe is basically consistent with the coupling vibration law of the top tension of the lifting pipe, indicates that the variation of the coupling tension largely depends on the relative motion displacement between the bottom of the lifting pipe and the mining vessel.

**Figure 16:**
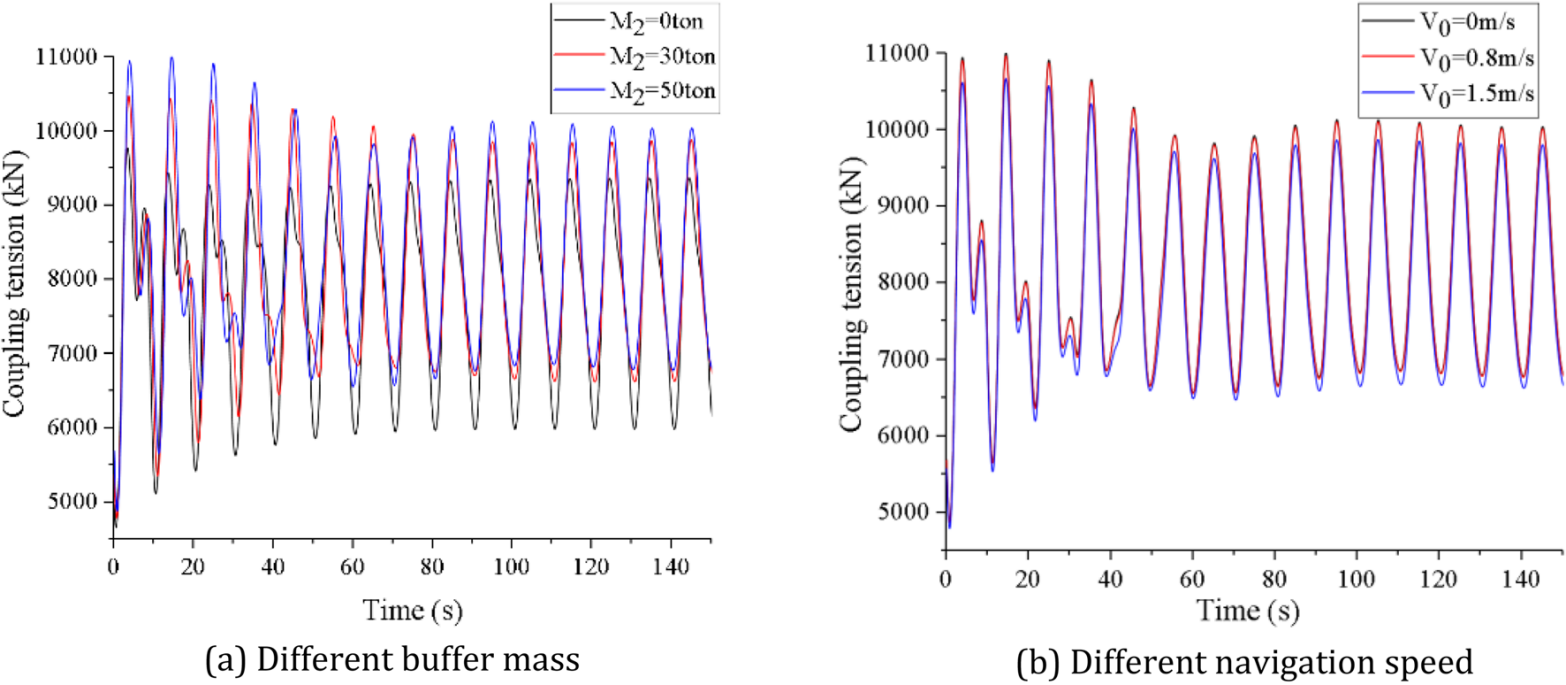
Time domain curve of coupling tension under different buffer mass and different navigation speed (created by Origin 2017 software, URL: https://www.originlab.com/).

Finally, we make simulation on the influence of the buffer mass and the vessel speed on the coupling tension. It is assumed that the wave direction is 90°, the buffer mass M_2_ is 0ton, 30ton and 50ton respectively, and the navigation speed V_0_ is 0 m/s, 0.8 m/s and 1.5 m/s respectively. OrcaFlex software is used to simulate the coupling tension under the above conditions in time domain. As shown in Fig. 16, the buffer mass and the vessel speed have little influence on the coupling action time, but have a greater influence on the coupling tension value. With the increase of buffer mass, the coupling tension oscillation balance axis moves up, and the maximum coupling tension value increases gradually. With the increase of navigation speed, the coupling tension oscillation balance axis moves down, and the maximum coupling tension value decreases gradually. When the buffer mass is 0ton and 50ton respectively, the maximum coupling tension values are 9771.07kN and 10,944.3kN respectively, the difference is 10.72%, which increases by 27.34% and 29.89% respectively compared with their static equilibrium values. When the navigation speed is 0 m/s and 1.5 m/s, the maximum coupling tension values are 10,944.3kN and 10,617.21kN respectively, and the difference is only 2.99%. It is not difficult to find that the coupling tension is more sensitive to the buffer mass than the navigation speed, and the increase of the buffer mass will aggravate the oscillation amplitude of the coupling tension.

**Figure 17:**
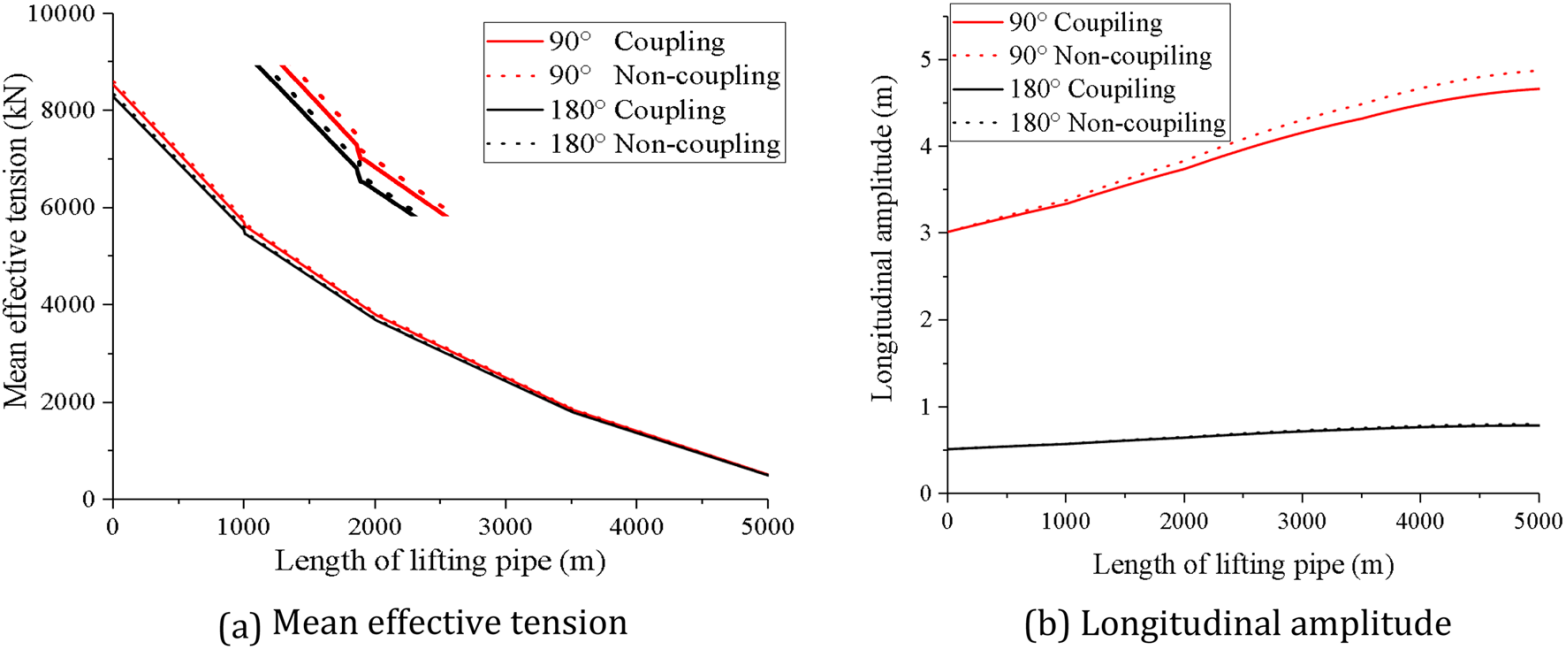
Dynamic response of lifting pipe along pipe length under different wave directions (created by Origin 2017 software, URL: https://www.originlab.com/).

The mean effective tension and longitudinal amplitude of the lifting pipe at different wave directions are simulated to illustrate the influence of coupling action on the dynamic responses of the lifting pipe along the pipe length. The results are compared with those of the pipeline without considering coupling action, as shown in Fig. 17.

As can be seen from Fig. 17, the dynamic responses of the lifting pipe at 90° wave direction is larger than that at 180° wave direction. When the wave direction is the same, the mean effective tension of the lifting pipe decreases step by step from top to bottom along the pipe length, and reaches the minimum at the buffer. At 1000 m, the mean effective tension changes abruptly due to the influence of the pump mass. Meanwhile, the longitudinal vibration amplitude of the lifting pipe along the pipe length presents a trend of small at the upper end and large at the lower end, and reaches the maximum at the buffer. Comparing the dynamic responses of the lifting pipe with and without coupling action, it can be found that when the wave direction is 90°, the mean effective tension at the top of the lifting pipe is 8530.77kN and 8601.87kN, respectively, with a difference of 0.83%, and the amplitude at the bottom of the lifting pipe is 4.67 m and 4.88 m, respectively, with a difference of 4.5%. When the wave direction is 180°, the mean effective tension at the top of the lifting pipe is 8285.60kN and 8344.56kN, respectively, with a difference of 0.71%, and the amplitude at the bottom of the lifting pipe is 0.79 m and 0.81 m, respectively, with a difference of 2.53%. The above results fully indicate that the wave direction affects the effect of the coupling action. After considering the coupling action, the dynamic response of the lifting pipe is weakened, but the coupling action has little effect on the mean effective tension and longitudinal amplitude along the pipe length.

## Conclusions

In this paper, a vessel-pipe coupling dynamic model of the deep-sea mining system, considering the ocean current, the motion of the mining vessel, the vibration of the lifting pipe and the coupling between the vessel and the pipe, is established. The hydrodynamic analysis of the mining vessel is carried out by using the finite element software AQWA, and the dynamic performances of the coupling system under different sea states and wave directions are simulated by software OrcaFlex. The vessel-pipe coupling model experiments are carried out, and the simulation results are in good agreement with the experimental results. The main conclusions are as follows:

1. The wave direction and sea state level have a great influence on the dynamic response of vessel-pipe coupling system. At 180° wave direction, the dynamic response is small, and the coupling action time is longer up to 300 s; at 90° wave direction, the dynamic response is significant, and the coupling action time is about 100 s. The difference of heave amplitude, maximum coupling tension, buffer amplitude and vessel-pipe coupling action time are 82.78%, 19.31%, 83.08% and 66.67% respectively under two wave directions of level 6 sea state. The motion response of the mining vessel is positively correlated with the sea state level, and the heave amplitude can reach 3.02 m at level 6 sea state. When the wave direction is 180°, the motion responses of the mining vessel are more significant under different sea states, and the differences of heave amplitude and pitch amplitude are 76.15% and 84.7% respectively. Therefore, the dynamic response of the coupling system can be effectively reduced by reasonably selecting the forward direction of the mining vessel and the operating sea state level in practical engineering.
2. The lifting pipe has a certain influence on the mining vessel motion, and the degree of influence is related to the wave direction. When the wave direction is 90°, the lifting pipe has little effect on the amplitude of the mining vessel motion, and the amplitude of the mining vessel motion with or without the lifting pipe is basically unchanged; When the wave direction is 180°, the heave amplitude and pitch amplitude of the mining vessel with lifting pipe are 31.11%, 33.33%, 33.85% and 1.4% lower than those of the mining vessel without lifting pipe under the level 4 and level 6 sea states, respectively. Therefore, when operating under the level 6 sea state and 90° wave direction, compensation technology should be considered to weaken the heave and pitch motion of the mining vessel, thereby reducing the dynamic response of the system.
3. The coupling effect has a significant impact on the time-domain dynamic response of the lifting pipe, especially in the initial movement stage, the axial tension and longitudinal vibration displacement of the lifting pipe have irregular oscillations, and the system coupling effect is significant. The maximum axial tension occurs at the top of the pipe, and the maximum vibration displacement occurs at the bottom of the pipe. When the sea state is level 6 and the wave directions are 90° and 180° respectively, the difference between the maximum coupling tension and the maximum steady-state tension is 8.6% and 3.33% respectively, and the difference between the maximum coupling vibration displacement and the maximum steady-state vibration displacement of the lifting pipe is 27.4% and 31.3% respectively. The results clearly show the necessity of coupling analysis, which can provide a very intuitive basis for the safety design of key parts of the system.
